# Arthropod Natural Enemies in Biological Control: A Systematic Bibliometric Analysis 2016–2025

**DOI:** 10.3390/insects17060609

**Published:** 2026-06-09

**Authors:** Shi-Jie Qi, Jie Wang, Jing-Juan Zhao, Chu-Fei Liu, Su Wang, Nicolas Desneux

**Affiliations:** 1 Institute of Data Science and Agricultural Economics, Beijing Academy of Agricultural and Forestry Sciences, Beijing 100097, China; qishijie18@163.com; 2 College of Life and Environmental Sciences, Hangzhou Normal University, Hangzhou 311121, China; 3 China Agricultural University Library, China Agriculture University, Beijing 100193, China; liuchufei@cau.edu.cn; 4 Key Laboratory of Natural Enemies Insects, Ministry of Agriculture and Rural Affairs, Institute of Plant Protection, Beijing Academy of Agriculture and Forestry Sciences, Beijing 100097, China; wangsu@baafs.net.cn; 5 Université Côte d’Azur, INRAE, CNRS, UMR ISA, 06000 Nice, France; nicolas.desneux@inrae.fr

**Keywords:** bibliometric analysis, biological control, natural enemies, predators, parasitoids, integrated pest management

## Abstract

Arthropod natural enemies—encompassing predators and parasitoids—form the backbone of sustainable agriculture, delivering irreplaceable ecosystem services via biological pest suppression. Driven by global demand for eco-friendly alternatives to synthetic pesticides, research in this domain has grown sharply over the past decade. Here, we report a systematic bibliometric analysis of 6515 Web of Science Core Collection papers focused on arthropod natural enemies in biological control (2016–2025), with the goal of charting the field’s intellectual structure. Performance metrics confirmed an initial rapid increase from 2016 to 2019 followed by a plateau and a slight rise in 2025, with the US, China, and Brazil dominating output. Keyword co-occurrence networks pinpointed core themes, including conservation biological control, predatory mites, and integrated pest management (IPM). Temporal trends further revealed a pivot toward applied work on invasive pest systems. Co-citation analysis uncovered six foundational research clusters, while bibliographic coupling of 2021–2025 papers uncovered five active emerging subfields: landscape ecology and habitat manipulation, tri-trophic interaction mechanisms, high-impact invasive pest biocontrol, non-target risk assessment for introduced agents, and fall armyworm integrated management. We synthesize cross-cutting implications and outline future priorities—including AI-enabled rearing systems, functional biodiversity boosting, climate adaptation, and multifunctional landscape tuning. By consolidating historical progress and forward-looking directions, this framework empowers researchers, extension practitioners, and policymakers to scale sustainable pest management worldwide.

## Simple Summary

We present a bibliometric analysis of global arthropod natural enemy research in biological control with 10-year (2016–2025) publications, drawing on 6515 Web of Science Core Collection publications. The field expanded initially rapidly then stabilized, with 684 publications in 2025, with the United States, China, and Brazil emerging as leading contributors. Core inquiry centered on conservation biological control, predatory mites, and invasive pest suppression, while five burgeoning subfields including landscape ecology and fall armyworm management, came into focus. Forward-looking priorities include AI-powered natural enemy rearing, climate resilience, and multifunctional landscape design. This analysis distills global research trends and delivers an actionable roadmap for advancing sustainable pest management across continents.

## 1. Introduction

Global food security is increasingly challenged by a growing population, climate change, and the demand for sustainable agricultural practices. Conventional pest management, heavily reliant on synthetic chemical pesticides, has led to numerous negative consequences, including the development of pest resistance, negative impacts on non-target organisms, environmental contamination, and risks to human health [1,2,3]. In response, there is a global shift towards more ecologically sound approaches, with biological control emerging as a cornerstone of integrated pest management (IPM) [4,5,6].

Biological control, or biocontrol, utilizes living organisms-natural enemies to suppress pest populations below economically damaging levels. These natural enemies, including predators, parasitoids, and pathogens, provide vital ecosystem services that enhance crop resilience and reduce reliance on chemical inputs [7,8,9,10,11]. The use of arthropod natural enemies has a long and successful history, ranging from classical biological control programs to augmentative releases in high-value crops [12,13]. The past decade (2016–2025) has witnessed a surge in research activity, driven by advances in molecular biology, chemical ecology, and landscape ecology, providing new insights into the complex interactions within agroecosystems [14,15,16].

While literature output has surged, a holistic, macro-scale synthesis that systematically charts the research landscape, pinpoints dominant trends, and identifies cutting-edge frontiers remains missing. Conventional narrative reviews are often narrow in scope and fail to capture the full breadth and evolutionary trajectory of the field. In contrast, bibliometric analysis provides a robust, data-driven framework for interrogating large scientific datasets, revealing hidden structural patterns, dynamic shifts, and nascent research themes [17,18].

This study aims to fill this gap by conducting a systematic bibliometric analysis of the global research on arthropod natural enemies in biological control over the last decade (2016–2025). The objectives are: (1) to analyze the performance of research activity, including publication trends, key journals, influential authors, countries, and institutions; (2) to identify the main research hotspots and thematic clusters through keyword co-occurrence analysis; (3) to trace the intellectual foundation of the field via co-citation analysis; and (4) to discover emerging and future research trends through bibliographic coupling. By synthesizing these findings, this paper provides a valuable roadmap for researchers, practitioners, and policymakers, fostering a deeper understanding of the field and guiding future efforts towards sustainable pest management.

## 2. Materials and Methods

We adopted a systematic bibliometric design to examine global literature on arthropod natural enemies in biological control. Methodological choices adhered to established bibliometric standards [19,20] and the PRISMA guidelines for systematic reviews [21], guaranteeing transparency and replicability.

### 2.1. Data Source and Search Strategy

The Web of Science Core Collection (WoS, https://clarivate.com/webofsciencegroup/solutions/web-of-science-core-collection/, accessed on 20 January 2026) was selected as the data source due to its comprehensive coverage of high-impact, peer-reviewed journals across the agricultural and biological sciences. The search was designed to capture publications focused on the use of arthropod natural enemies for biological control. The search was conducted using the topic query “TS = (((natural enemy OR natural enemies OR natural insect OR natural insects OR predatory natural enemy OR predatory natural enemies OR Polyphagous predator OR parasitoid OR Polyphagous predators OR parasitoids OR parasitic wasp OR parasitic wasps OR predatory mite OR ladybird beetle OR ladybugs OR ladybug OR ladybird OR lacewing OR green lacewing OR hoverfly OR hover fly OR hoverflies OR flower bug OR minute pirate bug OR mirid predatory bug OR predatory mirid OR trichogramma OR ichneumonidae OR pteromalidae OR braconidae OR coccinellidae OR chrysopa OR Anastatus OR orius OR aphidius OR amblyseius OR nesidiocoris tenuis OR phytoseiulus persimilis OR amblyseius OR neoseiulus californicus OR encarsia Formosa OR Harmonia axyridis OR Orius sauteri OR Dastarcus helophoroides OR Bethylus OR Chouioia cunea OR Eocanthecona furcellata OR Rhinocoris rubricus OR Picromerus lewisi OR Arma chinensis OR Diglyphus isaea OR Hypoaspis miles OR Aphidoletes aphidimyza OR Aphelinidae OR Trichopria drosophilae) and (biologic* control)) NOT (animal OR poultry OR fowl OR livestock OR human OR female OR body OR medicine OR medical OR inflammat* OR mice OR tumour OR vaccine OR cancer OR leukemia OR liver OR ocean OR sea OR marine OR immune* OR gut))” covering publications from 1 January 2016 to 31 December 2025 (topic searches encompass the Title, Abstract, Author Keywords, and Keywords Plus fields, maximizing the retrieval of relevant literature). The search was executed on 20 January 2026, yielding a total of 6515 publications after removing unrelated ones manually.

An initial search of the Web of Science database yielded 6683 records. After removing 6 duplicates, 108 conference abstracts or letters to journal editors, and 9 editorial materials, 6560 records remained. Following the exclusion of 27 records due to missing abstracts or keywords, 6533 records were screened by title and abstract. Ultimately, 18 records were excluded for not meeting the inclusion criteria, resulting in a final inclusion of 6515 studies for this review (Figure S1).

### 2.2. Cluster Theme Derivation for Bibliographic Coupling

To assign meaningful labels to the bibliographic coupling clusters, we followed a three-step procedure. First, for each cluster, we extracted the 10 most frequent author keywords from all documents in the cluster. Second, two authors (S.Q. and J.W.) independently inspected the titles and abstracts of the 20% most centrally connected documents (ranked by total link strength within the cluster). Third, the three co-authors (S.W., N.D., J.Z.) met to reach a consensus on the cluster label. Disagreements were resolved by discussion. This method ensures that the cluster labels reflect both the quantitative keyword patterns and the substantive content of the most influential papers in each cluster.

### 2.3. Data Analysis

Full publication records and cited references were exported to VOSviewer v.1.6.20 and CiteSpace 6.3.R1 for network and temporal analyses. Publication and citation trends were summarized via Web of Science in-built analytics and Microsoft Excel. This included analyses of annual publication output, most prolific journals, most cited articles, most productive and influential authors, leading institutions and countries, and major funding agencies. To identify research hotspots, a keyword co-occurrence network was constructed using VOSviewer v.1.6.20 with a minimum threshold of 10 occurrences per keyword (a thesaurus file was used to merge synonyms and singular/plural variants). For co-citation analysis of cited references, a minimum of 20 citations per cited reference was applied, and the resulting network was clustered using the default algorithm in VOSviewer v.1.6.20. To detect emerging research fronts, bibliographic coupling analysis was performed on documents published between 2021 and 2025, requiring a minimum of 5 common references for two documents to be coupled. Sensitivity analyses were conducted by varying these thresholds (keyword minimum: 5, 10, 15; co-citation minimum: 20, 30, 50; coupling minimum: 3, 5, 8). The main cluster structures remained stable across reasonable thresholds; minor variations did not alter the interpretation of the results.

## 3. Results

### 3.1. Performance Metrics

#### 3.1.1. Temporal Progression of Publications

**Figure 1:**
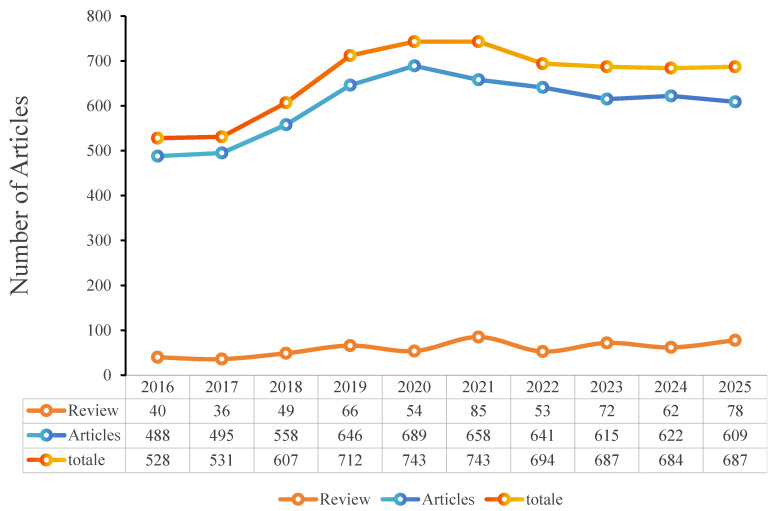
The yearly publication counts in the study domain of arthropod natural enemies in biological control.

The annual number of publications on arthropod natural enemies in biological control increased rapidly from 528 in 2016 to a peak of 743 in 2020, then stabilized at approximately 687–694 papers per year between 2021 and 2025 (Figure 1), with a 10-year total of 6515 publications. This pattern—rapid growth followed by a plateau—may reflect the maturation of the field or external factors such as the COVID-19 pandemic, but the sustained output of nearly 700 papers annually indicates continued global investment in this research area.

#### 3.1.2. Publishers and Journals

The 6515 publications were distributed across 732 journals. Biological Control leads the field with 455 articles, underscoring its role as the primary outlet for specialized research. Journal Insects follows with 430 articles, reflecting the open-access model’s growing influence. Other key journals include BioControl (208), Journal of Economic Entomology (196), Pest Management Science (195), and Journal of Pest Science (186), indicating a broad distribution of research across applied entomology and pest management periodicals (Table S1).

#### 3.1.3. Reviews and Research Articles

The influence of a publication is often measured by its citation count. These highly cited works address broad and fundamental topics. For review publications, habitat fragmentation and management [22,23], tri-trophic interactions mediated by plant volatiles [23], and reviews on the management of invasive pests like the tomato leafminer, *Tuta absoluta* (Lepidoptera: Gelechiidae) [24], the brown marmorated stink bug, *Halyomorpha halys* (Hemiptera: Pentatomidae) [25], and the harlequin ladybird, *Harmonia axyridis* [26] are cornerstone references (Table 1). The most highly cited original research on arthropod natural enemy biological control focuses on key themes (Table 2). Landscape ecology examines how landscape composition and non-crop habitats affect pest suppression [2,3,27]. Habitat management tests conditions for flower strips or natural habitat to enhance biocontrol [28,29,30]. Collectively, these studies mark a shift from descriptive surveys to mechanistic, landscape-based, and practice-oriented research.

Based on the top 10 highly cited publications, research on biological control using arthropod natural enemies has evolved toward landscape-driven mechanisms, invasive pest management, and scalable application (Table 1 and Table 2). Landscape composition and configuration strongly influence predator-pest interactions and natural pest control, although the direction and magnitude of effects can be context-dependent (e.g., Karp et al., 2018 [3]); nevertheless, a quantitative synthesis by Rusch et al. [27] found that landscape simplification reduces natural pest control by an average of 46%. Recent studies address context dependency and propose mechanistic hypotheses to explain inconsistent enhancement by natural habitats (e.g., Tscharntke et al. [28], who outlined five hypotheses for when natural habitat fails to support biocontrol), establishing landscape ecology as a core framework. Meanwhile, biological control using invertebrates and microorganisms offers extensive new opportunities, with growing focus on promoting real-world adoption [31]. High-impact research targets major invasive pests including *T. absoluta*, and *Hal. halys*, clarifying invasion dynamics and sustainable management strategies [24,25]. Research on the Asian lady beetle, *Harmonia axyridis* (Coleoptera: Coccinellidae) highlights non-target risks and improves risk assessment for introduced natural enemies [26]. Cross-scale studies across Europe advance functional biodiversity and agroecosystem service management [2]. Innovative robotic technology with switchable electrostatic adhesion emerges as a new frontier. Collectively, the field integrates landscape ecology, invasion biology, and applied entomology, shifting toward context-aware, risk-smart, landscape-based, and practically adoptable biological control.

#### 3.1.4. Authors Who Produce and Have Influence

The field drew contributions from 20,344 authors; applying Price’s law (≥8 publications, ≥48 citations) yielded 331 core researchers. Nicolas Desneux led both productivity (102 articles) and citation impact (4046 total citations), with Antonio Biondi, Alberto Urbaneja, and Marc Kenis also ranking among the most prolific and influential scholars (Table 3). The author collaboration network shows Desneux as a central hub, facilitating extensive international and interdisciplinary collaboration (Figure S2).

#### 3.1.5. Affiliations of the Authors

The United States Department of Agriculture (USDA) leads with 537 articles, followed by French institutions INRAE (363) and CNRS (243). The prominence of the University of California System, the Chinese Academy of Agricultural Sciences (CAAS), and EMBRAPA (Brazil) highlights the global distribution of research capacity, with strong representation from North America, Europe, South America, and Asia (Table S2).

#### 3.1.6. Geographical Distribution of Contributing Countries

The United States is the most prolific country, contributing 1604 publications (24.24% of total), followed by China (1116; 16.87%) and Brazil (770; 11.64%) (Table S3, Figure S3). The high total link strength for the USA and France indicates their central role in international collaborative networks. China’s strong performance, particularly with an average publication year of 2021.35, points to its rapidly growing and recent contribution to the field.

To account for differences in population size and economic capacity, we also calculated publications per million inhabitants and publications per billion USD GDP (PPP) for the top 10 most productive countries (Table S4). Among these countries, Australia (11.97 publications per million) and Spain (8.79) showed the highest per-capita outputs, followed by France (7.87) and Italy (6.71). In contrast, China (0.79) and Germany (3.47) exhibited the lowest per-capita values despite their high absolute publication counts. When normalized by GDP (PPP), Australia (0.167 publications per billion USD) and Brazil (0.163) ranked highest, indicating strong national research investment in biological control relative to their economic size. Spain (0.151) and France (0.126) also showed substantial GDP-normalized outputs, while the United States (0.055) and China (0.029) had lower ratios due to their larger economies. These normalized indicators provide a complementary perspective on national research commitment beyond absolute publication counts.

#### 3.1.7. Financial Institutions Providing Funds

Funding for this research is global, with major agencies from China, Brazil, the USA, and Europe (Table S5). The National Natural Science Foundation of China (NSFC) is the top funder (479 articles; 7.24%), followed by Brazilian agencies CNPQ (390) and CAPES (350). This aligns with the high publication output from these countries and underscores their national commitment to advancing biological control research.

To compare funding intensity relative to economic size, we normalized the number of funded articles by national GDP (PPP). As shown in Table S6, Brazil exhibits the highest funding-to-GDP ratio among all countries, with CNPq contributing 82.3 funded articles per trillion USD GDP, CAPES 73.8, and the state-level agency Fapemig 23.0. The combined contribution of CNPq and CAPES (156.1 articles per trillion USD GDP) far exceeds that of any other national funding system, reflecting Brazil‘s exceptionally strong institutional commitment to biological control research relative to its economic size. Spain also demonstrates a remarkably high ratio (71.3 articles per trillion USD GDP through its national government funding), followed by the United Kingdom (UKRI: 25.0) and the United States (USDA: 11.6; NSF: 4.0). China’s ratios are more moderate (NSFC: 12.6; National Key R&D Program: 3.2), which is expected given its large economy. Several European countries, despite having moderate absolute funding counts, show GDP-normalized intensities comparable to or exceeding those of larger economies. These normalized indicators provide a complementary perspective on national research prioritization beyond absolute funding counts.

### 3.2. Research Hotspots

#### 3.2.1. Keyword Co-Occurrence Analysis

To identify the main research themes, a keyword co-occurrence analysis was performed on the 6515 publications (Figure S2). The network map reveals several distinct clusters. The most prominent keywords are biological control, natural enemy, management, biodiversity, and integrated pest management. The overlay of average publication year (Figure S3; numerical values for the top 30 keywords are provided in Table S7) shows that while terms like ‘biological control’ (average year 2018.2) and ‘parasitoid’ (2018.5) are more established, newer research hotspots include ‘conservation biological control’ (2020.3), ‘climate change’ (2021.8), and specific pest names like ‘*Tuta absoluta*’ (2020.7) and ‘*Halyomorpha halys*’ (2021.1), indicating a shift towards applied ecology and addressing specific invasive threats.

#### 3.2.2. Keyword Clustering Timeline

**Figure 2:**
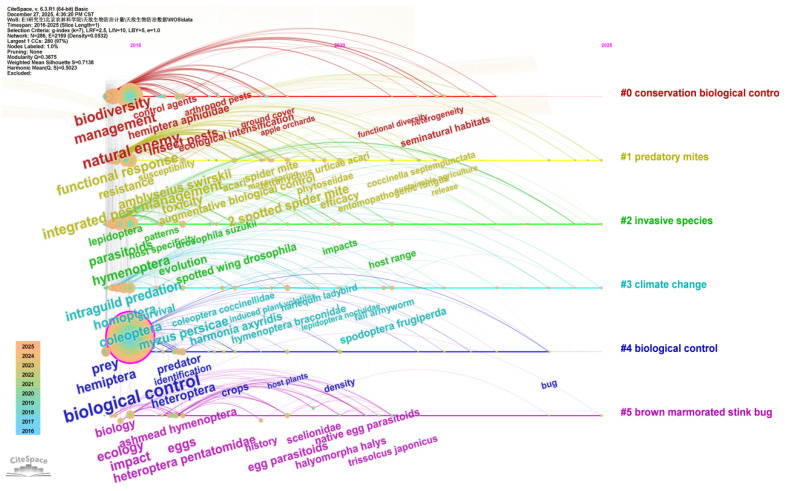
The keyword clustering timeline in the scientific field of biological control with arthropod natural enemies (Based on the literature data retrieved from Web of Science, this study adopted CiteSpace 6.3.R1 software for bibliometric analysis. The time span was set from 2016 to 2025 with a one-year time slice. The g-index with k = 7 was selected as the data screening criterion, and the network pruning operation was not conducted. A total of 286 nodes and 2169 links were generated, with a network density of 0.0532. The largest connected component contained 97% of all nodes, indicating strong internal relevance of the research field. The modularity Q value was 0.3875 and the weighted mean silhouette S value was 0.7138, both of which indicated that the clustering results were reasonable, hierarchical and highly consistent within each cluster. The harmonic mean of Q and S was 0.5023, verifying the reliability and validity of the visual clustering results).

The timeline-pan in CiteSpace 6.3.R1 was employed to analyze the temporal evolution of various research themes. Additionally, the burst detection algorithm was applied to identify keywords that showed a sudden and sharp surge in frequency over specific periods. By integrating the Burst Detection function, the burst keywords associated with the temporal evolution of these research themes can be clearly identified (Figure 2).

The bibliometric analysis covered the period from 2016 to 2025 with a slice length of 1 year. Using the g-index algorithm, the pruning method was set to “None”. The optimal number of clusters identified was 7; however, to ensure clustering quality, the software automatically merged one cluster containing only 5 papers, resulting in a final configuration of 6 clusters. The network comprised 286 nodes and 2169 edges. The Largest Connected Component (LCC) accounted for 97% of the network, indicating strong overall connectivity The Weighted Mean Silhouette value was 0.7138, which measures the homogeneity and consistency of cluster content—values closer to 1 indicate superior clustering quality. The Harmonic Mean value (combining Modularity Q and Silhouette S) was 0.5023, serving as a comprehensive evaluation of both network structure and clustering quality; a value exceeding 0.5 indicates statistical significance.

A detailed quantitative analysis was conducted on the burst occurrence of keywords, and a keyword burst chart was generated. In this study, the burst chart is sorted by the starting year of burst. Red lines represent the burst periods of each keyword and the time segments during which research hotspot has been sustained in the field of arthropod natural enemy biological control (Figure 2).

Chronologically, research can be divided into four stages (Figure 2). 2016–2018: Initial stage—hotspots of basic ecological elements. Core burst keywords were species richness (burst strength: 12.99), cereal aphids (12.52), beneficial insects (11.17). This stage was a concentrated burst period of fundamental research in the field, centered on ecological community structure (species richness), typical agricultural pests (cereal aphids), and natural enemy communities (beneficial insects), reflecting core themes that have remained central to biological control for decades. 2017–2019: Stage of control technology and target expansion. Core burst keywords were suppression (10.23), marmorated stink bug (8.41), augmentative biological control (8.37). The focus shifted from basic elements to technical application, concentrating on pest control tactics (suppression, augmentative biological control) while expanding to new core pests (marmorated stink bug). 2020–2022: Stage of research on major invasive pests and their impacts. Core burst keywords were the fall armyworm, *Spodoptera frugiperda* (Lepidoptera, Noctuidae) (10.12), *Hal. halys* (9.99), and impacts (9.31). Research targets converged on major invasive pests (marmorated stink bug, fall armyworm), with concurrent focus on ecological and agricultural impacts caused by pests, marking an application-oriented hotspot phase. 2022–2025: Stage of habitat manipulation and sustained hotspots. Core burst keywords were seminatural habitats (11.01), egg parasitoids (8.69), and fall armyworm (9.81). An ecological perspective of habitat manipulation (seminatural habitats) was introduced, while research on major pests (fall armyworm) and natural enemy resources (egg parasitoids) continued, representing an integration of ecological regulation and target species.

The western flower thrips, *Frankliniella occidentalis* (Thysanoptera: Thripidae) showed sustained bursts from 2016 to 2020, serving as a cross-stage core pest. *Spodoptera frugiperda* (fall armyworm) exhibited sustained bursts from 2021 to 2025, making it the most active research target at present (Figure S5).

### 3.3. Co-Citation Analysis: Identification of Prominent Research Clusters and Highly Cited Papers in the Field Under Study

Co-citation analysis of cited references identified six major clusters representing the intellectual foundations of the field (Figure S6). These clusters include key work on conservation biological control (#0), the biology and use of specific natural enemy groups like predatory mites (#1), the ecology of invasive species like *Har. axyridis* (#2) and *Hal. halys* (#5), the impacts of climate change on biocontrol (#3), and the application of biological control against major pests like *T. absoluta* (#4) [26,27,28].

**Figure 3:**
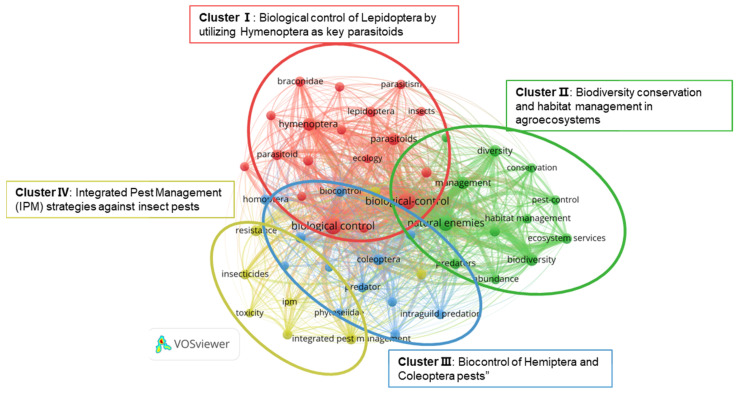
Co-citation clustering: major research clusters of arthropod natural enemies in biological control.

With co-citation network analysis, data clustering has revealed four distinct clusters in the field of biological control with arthropod natural enemies (Figure 3). Cluster 1 focuses on the biological control of Lepidoptera and other insect pests by utilizing Hymenoptera (primarily Braconidae) as key parasitoids, with core research emphases on the mechanisms of parasitism, the ecological characteristics of parasitoid insects, and the practical application of parasitoid-based biological control strategies for insect pests. Cluster 2 centers on biodiversity conservation and habitat management in agroecosystems, exploring the regulation of natural enemy abundance and diversity through habitat management measures, and revealing the role of natural enemies (especially predators) in pest control and the provision of related ecosystem services for sustainable pest control. Cluster 3 is dedicated to the biocontrol of Hemiptera and Coleoptera pests (mainly mite and insect pests), with a focus on Phytoseiidae as dominant predatory natural enemies, and core research on predator-prey interactions, the phenomenon and impacts of intraguild predation among predators, and the application of predatory biocontrol agents in pest management. Cluster 4 focuses on integrated pest management (IPM) strategies against insect pests, with core research on the compatibility of insecticides with biological control, including insecticide resistance in pests, the toxicity characteristics of insecticides (including to non-target organisms), and the rational selection and application of insecticides in the framework of IPM to achieve sustainable and effective pest control.

### 3.4. Using Bibliographic Coupling Analysis to Discover Emerging Research Areas

**Figure 4:**
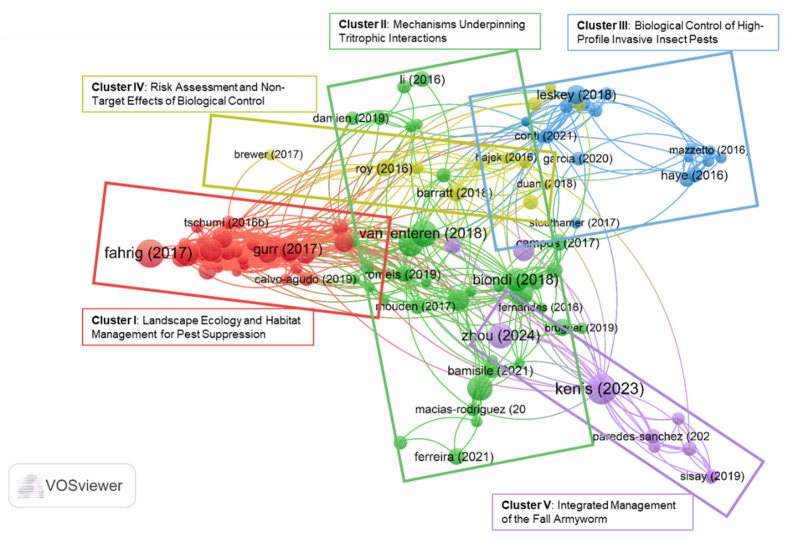
Bibliographic coupling clustering of documents published between 2021 and 2025 in the field of arthropod natural enemies in biological control. Each node represents a document. The label shows the first author’s surname and the publication year of that document (e.g., “Fahrig (2017) [22]”). Node colors indicate cluster membership (as identified in the analysis). Node size is proportional to the document’s total link strength (the sum of coupling strengths with all other documents in the network). Clusters are labeled based on their dominant research themes.

Bibliographic coupling was performed on the 2847 documents published between 2021 and 2025 (the most recent five years of the study period). A minimum of five common references was required for a document to be included (References used in analysis are available in Table S8), and the analysis yielded five major clusters (Figure 4). In Figure 4, each node represents a document, labeled by the first author’s surname and its publication year. The quantitative characteristics of each cluster are summarized in Table 1 below. For each cluster, we report the number of documents, average publication year, silhouette value (a measure of cluster homogeneity, with values > 0.7 indicating strong internal consistency), and the top five theme keywords derived from author keywords. Table S9 (Supplementary Materials) lists the five most central papers (by local citation count) for each cluster, along with their total link strengths (Table S9). These are described below.

- Cluster I: Landscape Ecology and Habitat Management for Pest Suppression

Cluster 1 comprises 43 documents with an average publication year of 2017.86 and a very high silhouette of 0.977, indicating excellent cluster homogeneity. The dominant theme keywords (pest regulation, agroforestry, biodiversity, ecosystem services, landscape ecology) reflect a sustained focus on how landscape composition and configuration affect natural pest suppression. Core papers in this cluster include landscape-scale syntheses on habitat management and conservation biological control (see Table S9). Landscape-scale habitat configuration and diversification enhance biological control services through natural enemy conservation and functional connectivity. This cluster focuses on how the structure and composition of agricultural landscapes influence natural enemies and the ecosystem service of pest control. A central theme is testing the assumption that non-crop habitat universally benefits pest control. Karp et al. [3] found that while landscape composition explains variation within studies, there is no consistent cross-study trend, with pest control sometimes increasing and sometimes decreasing with more non-crop habitat. However, other large-scale syntheses provide more nuanced support. Martin et al. [2] demonstrated that landscape configuration (edge density) interacts with habitat proportion, showing that high edge densities can significantly boost pollinator and natural enemy abundances, leading to 1.4-fold improvements in pest control. Similarly, Rusch et al. [27] provided quantitative evidence that landscape simplification (high proportion of cultivated land) consistently reduces the level of natural pest control by an average of 46%. Fahrig [22] contributed a foundational perspective by reviewing fragmentation studies and finding that 76% of significant responses to habitat fragmentation per se (independent of habitat amount) were positive, challenging the assumption that smaller patches have lower conservation value. Although Fahrig’s review focuses on landscape fragmentation broadly, its findings on predator–prey and host–parasitoid dynamics have direct implications for biological control by natural enemies. This supports the idea that land sharing can provide high ecological value. Several studies explore the effectiveness of specific interventions. Tscharntke et al. [28] outlined five hypotheses for when natural habitat fails to support biocontrol, such as when it acts as a pest source or when agricultural practices counteract enemy establishment. Begg et al. [7] synthesized this complexity into a conceptual model, arguing that while conservation measures often support natural enemy populations, the key limitation is frequently a failure to direct these services towards achieving specific pest suppression goals.

- Cluster II: Mechanisms Underpinning Tri-trophic Interactions

Cluster 2 contains 38 documents (average year 2018.39, silhouette 0.899). Keywords such as “integrated pest management,” “indirect defense,” and “plant volatiles” indicate that this cluster focuses on the chemical and biological mechanisms mediating interactions among plants, herbivores, and natural enemies. Research in this area emphasizes herbivore-induced plant volatiles (HIPVs) and their potential for enhancing biological control. The role of plant volatiles, host plant resistance, and bottom-up effects in mediating tri-trophic interactions between crops, herbivorous pests, and their natural enemies. This cluster investigates the chemical and biological mechanisms that govern interactions within the food chain, focusing on how plants mediate pest control. Turlings and Erb [23] reviewed the ecological and mechanistic roles of herbivore-induced plant volatiles (HIPVs) in attracting natural enemies, highlighting gaps in knowledge that hinder their exploitation for crop protection. Aartsma et al. [39] further explored this by discussing how the “volatile mosaic” at a landscape scale influences parasitoid host-location, a crucial but under-researched area for sustainable pest management. The impact of host plants extends to the bottom-up forces acting on pests. Han et al. [40] summarized how practices like irrigation, fertilization, and crop resistance trigger bottom-up effects that can be leveraged for integrated pest management (IPM). Vidal and Murphy [30] found that top-down forces from natural enemies are generally stronger than bottom-up forces from host plants on herbivorous insect fitness. The impact of climate change on these delicate tri-trophic interactions is a growing concern. Tougeron et al. [41] discussed how climate-induced phenological mismatches between hosts and parasitoids could disrupt biological control services. Finally, the nutritional ecology of natural enemies is a key component. Benelli et al. [42] reviewed the importance of adult diet, including nectar and honeydew, for parasitoid fitness and reproductive success, highlighting honeydew as a crucial but often overlooked carbohydrate source that can shape interactions with other arthropods like ants.

- Cluster III: Biological Control of High-Profile Invasive Insect Pests

Cluster 3 includes 18 documents (average year 2017.92, silhouette 0.721—still acceptable as >0.7). The keywords “biocontrol,” “chemical ecology,” “Hemiptera,” “invasive species,” “landscape management,” “pre-emptive,” and “classical biological control” reveal a dual focus on the chemical ecology of invasive pests and proactive biocontrol strategies. This cluster is notable for introducing the concept of pre-emptive risk assessment for classical biological control agents. Research on the biological control of major invasive pests, focusing on the role of natural enemies, particularly parasitoids, in regulating populations of the spotted-wing drosophila, *Drosophila suzukii* (Diptera: Drosophilidae) and *Hal. halys*. For *Hal. halys*, research consistently points to the inadequacy of native natural enemies in invaded regions. Abram et al. [43] synthesized 98 datasets and confirmed that while egg mortality from native enemies can reach 5–25%, it is still far below levels needed for control. This has led to a strong focus on classical biological control using the Asian egg parasitoid *Trissolcus japonicus*. Leskey and Nielsen [25] provided a comprehensive overview of *Hal. halys* IPM, noting that understanding its dispersal from wooded habitats and the identification of its aggregation pheromone have been key to developing behavioral controls alongside biological control efforts. Since its 2008 invasion of Europe and North America, *D. suzukii* has become a major global fruit pest, driving extensive research into sustainable controls [44]. Augmentative releases of the samurai wasp, *Trichopria drosophilae* (Hymenoptera: Scelionidae) have proven effective in reducing early-season populations [45]. These integrated approaches are vital for sustainable management of this pervasive pest.

- Cluster IV: Risk Assessment and Non-Target Effects of Biological Control

Cluster 4 is a smaller cluster (12 documents, average year 2018.72, silhouette 0.869). The keywords “invasiveness,” “biotic resistance,” “establishment,” and “risk assessment” indicate that this cluster critically examines the potential negative impacts of introduced natural enemies, including non-target effects and the factors that determine successful establishment. This research area has gained prominence following high-profile invasions. Examining the dual role of introduced natural enemies as both successful biocontrol agents and potential invasive species, including non-target effects, regulatory challenges, and paradigm shifts in risk assessment. This cluster takes a critical look at biological control itself, examining its history, its risks, and the ecological complexities it introduces. A prominent case study is the harlequin ladybird (*Har. axyridis*). Roy et al. [26] reviewed its global invasion, detailing how this intentionally introduced biocontrol agent became a pest itself, threatening biodiversity through competition and predation, and serving as a model for understanding invasion biology. Barratt et al. [35] identified the main limitations to biocontrol uptake, including risk-averse regulations and bureaucratic barriers, and called for better communication of its successes to stakeholders. This tension between benefits and risks is the central theme of a paradigm shift described by Heimpel and Mills [46].

- Cluster V: Integrated Management of the Fall Armyworm

Cluster 5 is the smallest but most recent cluster (10 documents, average year 2019.72, silhouette 0.891). Keywords include “biological control,” “egg parasitism,” “fall armyworm,” “invasive species,” “maize,” “*Spodoptera frugiperda*,” and “*Telenomus remus*.” This cluster reflects the urgent global research response to the rapid spread of fall armyworm across Africa, Asia, and Australia, with a strong emphasis on native egg parasitoids and sustainable IPM packages suitable for smallholder farmers. The rapid global invasion of the fall armyworm (FAW), its impact, and the urgent development of sustainable management strategies, with a strong emphasis on biological control and IPM. This cluster is dedicated to the fall armyworm (*S. frugiperda*), a recent and devastating invasive pest that has spread across Africa, Asia, and Australia. Kenis et al. [47] provided a comprehensive review of FAW, covering its biology, global spread, and all aspects of management, from chemical control and insecticide resistance to biological control and biopesticides. A significant portion of the research focuses on documenting and leveraging the pest’s natural enemies in its new ranges. Sisay et al. [48] conducted surveys in Ethiopia, Kenya, and Tanzania, discovering a complex of native parasitoids attacking FAW, providing a foundation for conservation and augmentative biological control. Finally, the challenge of implementing such complex strategies is addressed by Zhou et al. [49] in a broader review of IPM advances. They highlight the growing importance of conservation biological control, novel biopesticides, and targeted pesticide delivery systems, but also stress the need for transdisciplinary research, capacity building, and policy support for widespread adoption.

### 3.5. Sensitivity Analysis Results

Varying the minimum keyword occurrence from 10 to 5 or 15 changed the number of nodes but preserved the main clusters (conservation biocontrol, landscape, invasive pests). Co-citation clustering with minimum citations of 20, 30, or 50 yielded the same six clusters, though the ordering of cluster sizes changed slightly. Bibliographic coupling with a minimum of 3 or 8 common references instead of 5 produced similar clusters, but with more noise at the lower threshold and fewer documents at the higher threshold. The results presented are based on the default thresholds, which balanced cluster coherence and coverage.

## 4. Discussion

**Figure 5:**
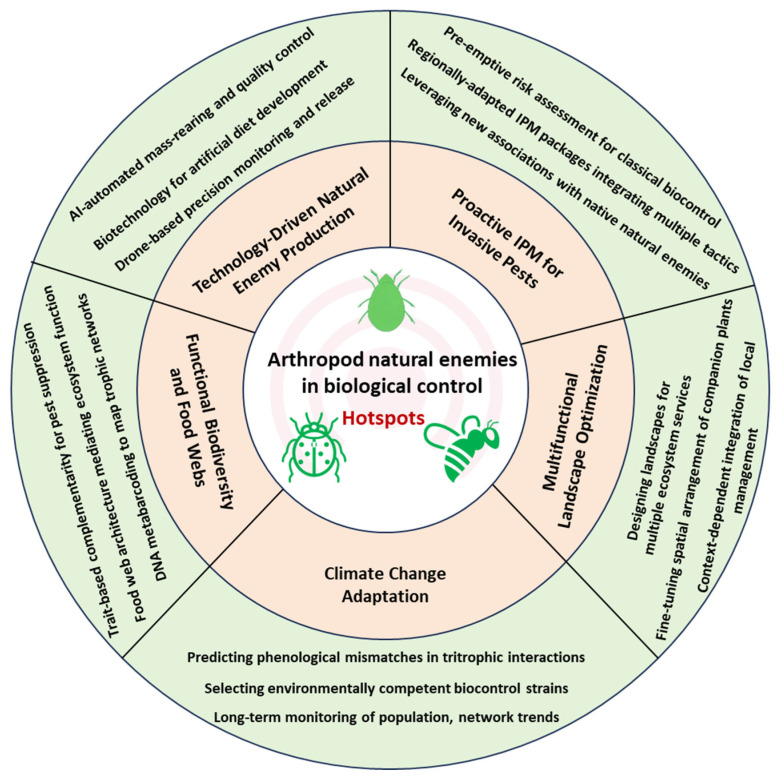
The research hotspots of arthropod natural enemies in biological control.

Before detailing these future directions, we first explicitly link each priority to the empirical results presented above (Figure 5). Priority 4.1 (AI-driven and biotechnological innovations) emerges from the burst keyword “robotic” (2022–2025) and from recently published (2024–2025) high-coupling-strength documents in our bibliographic coupling analysis that focus on artificial intelligence and automated natural enemy rearing. Priority 4.2 (functional biodiversity and food web dynamics) is directly rooted in co-citation Cluster II (tri-trophic interaction mechanisms) and bibliographic coupling Cluster II, both of which emphasize predator-parasitoid complementarity and multi-trophic networks. Priority 4.3 (multifunctional landscapes) corresponds to bibliographic coupling Cluster I (landscape ecology and habitat management) and co-citation Cluster #0 (conservation biological control), which together demonstrate how landscape configuration and habitat manipulation enhance pest suppression. Priority 4.4 (climate change adaptation) draws from co-citation Cluster #3, which specifically examines climate-induced phenological mismatches, and from the growing keyword node “climate change” in our co-occurrence network. Priority 4.5 (invasive pest IPM) is strongly supported by bibliographic coupling Clusters III (*Hal. halys* and *D. suzukii*) and V (fall armyworm), as well as by the sustained keyword bursts for *Hal. halys* and *S. frugiperda* shown in Figure 2. Thus, each of the five forward-looking priorities is a direct extension of the most active research clusters identified in our bibliometric analysis (Figure 5). The bibliometric dissection reveals a dynamic, fast-evolving field. Looking ahead, the domain is shifting decisively toward technology-enabled solutions, landscape-scale optimization, and a more nuanced molecular and functional understanding of biological control agents.

### 4.1. Ai-Driven and Biotechnological Innovations in Natural Enemy Production

Although artificial intelligence (AI) and robotics are not yet major themes in the mainstream literature of 2016–2025 (the keyword “robotic” has a low frequency and a short burst period), the most recent publications (2024–2025) as well as emerging technological advances suggest that this will become an important frontier. The integration of artificial intelligence, biotechnology, and automated platforms has the potential to transform the cost-efficient, high-quality mass-rearing and precision deployment of natural enemies. For example, deep-learning-based detection approaches enable rapid and accurate identification of natural enemy species, which is essential for quality control in mass-rearing facilities and for monitoring field releases [50]. Notably, this shift addresses one of the field’s most persistent bottlenecks: the high cost and labor demands of conventional natural enemy production. Future research will heavily leverage artificial intelligence (AI) to automate and optimize mass-rearing. Javed et al. [51] highlight the potential of AI and machine learning for precise environmental control, behavioral monitoring, and quality assurance in bio-factories. AI can improve key aspects of NEs production, including environmental control, behavioral monitoring, and quality assurance. This will be coupled with biotechnological advances in diet formulation to move away from reliance on live hosts. Wang et al. [52] propose a unified framework for developing accurate, affordable, and host-independent artificial diets using biotechnology, which is crucial for scaling up production. Beyond production, precision agriculture technologies, such as drones, will be key. Iost Filho et al. [53] discuss using sensing drones for pest hotspot detection and actuation drones for targeted releases of natural enemies, a significant step towards precision biocontrol. Furthermore, the fundamental understanding of the agents themselves is advancing through genomics and molecular biology. The molecular mechanisms of host manipulation by parasitoids, including the role of venom and poly-dnaviruses, are being unraveled [11,54]. This knowledge can be used to select for more efficient strains or develop novel control strategies [55]. The integration of these technologies aims to make biocontrol more predictable, scalable, and economically viable for large-scale agriculture, moving from a “cottage industry” to a high-tech sector [12].

### 4.2. Unraveling and Enhancing Functional Biodiversity and Food Web Dynamics

Future research should move beyond species counts to understand functional traits, ecological network structure, and complementarity among natural enemies for optimized pest suppression. Future research will deepen its focus on the quality of biodiversity, not just its quantity. The emphasis is on understanding how functional traits—such as hunting strategy, habitat domain, and diet breadth—determine the outcome of species interactions and pest control. Snyder [8] demonstrates that complementarity among natural enemies from different functional groups is key to maximizing pest suppression, a principle that needs to be applied in landscape management. Yang et al. [56] used path analysis to show how parasitoid-hyperparasitoid network structure (e.g., generality, vulnerability) mediates ecosystem functionality, highlighting the need to account for these multi-trophic interactions. A major methodological challenge is incorporating the vast diversity of parasitoids into these networks. Miller et al. [57] advocate for using DNA metabarcoding to construct detailed host-parasitoid networks, which can reveal cryptic interactions and assess ecosystem service provision more accurately. Future work will use these tools to design landscapes and management practices that explicitly encourage niche partitioning and dampen negative interactions like intraguild predation. This includes explicitly engineering fields to support complementary enemy guilds, as proposed by Snyder [8] (i.e., using habitat management to bring together natural enemies that fill different feeding niches), and understanding how factors like intercropping [58] shape the balance between predator and prey communities across different spatial and temporal scales.

### 4.3. Optimizing Multifunctional Landscapes for Resilient Pest Control

Future research should transition from simple habitat provision to designing multifunctional landscapes that integrate crop diversification, field margins, and soil management for resilient ecosystem services. While the importance of landscape complexity is well-established [2,27], the future lies in optimizing its specific components and their interactions. Research is shifting from asking if non-crop habitat works to how and when it can be optimized without yield penalties. This involves fine-tuning the spatial arrangement of features. For example, Wang et al. [59] show that the spatial configuration of companion plants (clustered vs. row intercropping) within fields significantly impacts pest control, a scale often overlooked. The goal is to design multifunctional landscapes that simultaneously support pest control, pollination, and other services. Future research will explore how different diversification strategies, such as strip intercropping [60] and increasing crop diversity [61], can be implemented within existing farming systems to enhance predator diversity and biological control. Crucially, this optimization must consider context-dependency. Karp et al. [3] found no consistent global trend for non-crop habitat, underscoring the need for predictive tools. Future efforts will aim to understand how local management (e.g., tillage) [28] and landscape features interact to modulate pest control, moving towards region-specific recommendations.

### 4.4. Climate Change Adaptation and Biocontrol Resilience

A key future direction is to assess and mitigate the impacts of climate change on the phenological synchrony, distribution, and efficacy of natural enemy-pest interactions. Climate change poses a fundamental threat to the reliability of biological control by disrupting the finely tuned relationships between pests and their enemies [62]. A major future research direction is understanding and predicting phenological mismatches. Tougeron et al. [41] highlight that differing responses to warming between trophic levels can disrupt host-parasitoid synchrony, potentially leading to pest outbreaks. Future work will need to model these complex outcomes under various climate scenarios. For arthropod natural enemies, research will focus on their capacity for plastic and evolutionary adaptation to new climatic conditions, including changes in diapause, voltinism, and thermal tolerance [41]. Long-term monitoring, like the 18-year study by Zhou et al. [49], which documented a decline in natural enemy abundance and network connectome, is crucial for detecting and understanding these macro-scale impacts of global change.

### 4.5. Integrated Pest Management for Emerging and Established Invasive Pests

Developing proactive, sustainable, and regionally-adapted IPM packages that integrate classical, augmentative, and conservation biological control for key invasive pests [63]. Research on invasive pests like the fall armyworm, the tomato pinworm, and the spotted-wing drosophila is shifting from emergency response to developing long-term, integrated strategies. For the fall armyworm, a major focus is on leveraging the new associations with native natural enemies in invaded region [47,64]. Future efforts will aim to enhance these native parasitoid populations through conservation and augmentative releases, rather than relying solely on chemical controls. The adventive establishment of specialized parasitoids presents new opportunities but also necessitates careful study of their non-target effects and integration into existing food webs [47]. A forward-looking concept is pre-emptive biocontrol, Avila et al. [65] propose a framework for conducting risk assessments for potential biocontrol agents before a high-risk pest arrives in a country, dramatically speeding up response times. For established pests like *T. absoluta*, the challenge is to move from chemical-heavy control to true IPM. Desneux et al. [5] and Wang et al. [66] highlight the need for regionally-adapted IPM packages that combine biological control, biopesticides, and cultural practices, while also addressing the economic barriers to adoption for growers. This includes exploring the dual role of agents like predatory mirids [67] and hoverflies [68,69,70] and integrating them into holistic management plans.

## 5. Conclusions

This 10-year systematic bibliometric analysis offers a complete portrait of the global research landscape for arthropod natural enemies in biological control. The field is marked by strong, consistent growth, with the US, China, and Brazil as leading contributors. The most striking finding is the intense and growing focus on a small number of high-impact invasive pests, the fall armyworm, the brown marmorated stink bug, and the tomato leafminer, which together dominate recent keyword bursts and bibliographic coupling clusters. Landscape ecology and tri-trophic interactions have emerged as core conceptual frameworks. The intellectual foundation, revealed through co-citation analysis, is built upon key concepts in conservation biological control, landscape ecology, and invasion biology. The most current and emerging research, identified via bibliographic coupling, demonstrates a clear evolution towards integrated, multi-tactic strategies that combine classical biological control, augmentative releases, and habitat management. Future research priorities identified from the latest literature include AI-enabled rearing systems, functional biodiversity enhancement, climate resilience, and multifunctional landscape design. However, these are still emerging and require further empirical validation. By addressing these challenges, the scientific community can ensure that arthropod natural enemies play a central and expanding role in creating a more sustainable and food-secure future.

## Figures and Tables

**Table 1:** Top ten most referenced review publications in the field of biological control with arthropod natural enemies.

Title	Year	Authors	Journal	Citation	Reference
Ecological Responses to Habitat Fragmentation Per Se	2017	Fahrig, L	Annual Review of Ecology, Evolution, and Systematics	947	[22]
Biological control using invertebrates and microorganisms: plenty of new opportunities	2018	van Lenteren, JC et al.	BioControl	753	[31]
Tritrophic Interactions Mediated by Herbivore-Induced Plant Volatiles: Mechanisms, Ecological Relevance, and Application Potential	2018	Turlings, TCJ & Erb, M	Annual Review of Entomology	720	[23]
Ecology, Worldwide Spread, and Management of the Invasive South American Tomato Pinworm, *Tuta absoluta*: Past, Present, and Future	2018	Biondi, A et al.	Annual Review of Entomology	663	[24]
Habitat Management to Suppress Pest Populations: Progress and Prospects	2017	Gurr, GM et al.	Annual Review of Entomology	583	[32]
Impact of the Invasive Brown Marmorated Stink Bug in North America and Europe: History, Biology, Ecology, and Management	2018	Leskey, TC & Nielsen, AL	Annual Review of Entomology	387	[25]
The harlequin ladybird, *Harmonia axyridis*: global perspectives on invasion history and ecology	2016	Roy, HE et al.	Biological Invasions	341	[26]
Plant Defense against Herbivorous Pests: Exploiting Resistance and Tolerance Traits for Sustainable Crop Protection	2016	Mitchell, C et al.	Frontiers in Plant Science	324	[33]
Research trends in ecosystem services provided by insects	2018	Noriega, JA et al.	Basic and Applied Ecology	314	[34]
The status of biological control and recommendations for improving uptake for the future	2018	Barratt, BIP et al.	BioControl	299	[35]

**Table 2:** Top ten most referenced research publications in the field of biological control with arthropod natural enemies.

Title	Year	Authors	Journal	Citation	Reference
Crop pests and predators exhibit inconsistent responses to surrounding landscape composition	2018	Karp, DS et al.	PNAS	543	[3]
The interplay of landscape composition and configuration: new pathways to manage functional biodiversity and agroecosystem services across Europe	2019	Martin, EA et al.	Ecology Letters	532	[2]
When natural habitat fails to enhance biological pest control—Five hypotheses	2016	Tscharntke, T et al.	Biological Conservation	498	[28]
Agricultural landscape simplification reduces natural pest control: A quantitative synthesis	2016	Rusch, A et al.	Agriculture Ecosystems & Environment	496	[27]
Bottom-up vs. top-down effects on terrestrial insect herbivores: a meta-analysis	2018	Vidal, MC & Murphy, SM	Ecology Letters	201	[30]
Give predators a complement: Conserving natural enemy biodiversity to improve biocontrol	2019	Snyder, WE	Biological control	179	[8]
Tailored flower strips promote natural enemy biodiversity and pest control in potato crops	2016	Tschumi, M et al.	Journal of Applied Ecology	173	[29]
Nectar accessibility determines fitness, flower choice and abundance of hoverflies that provide natural pest control	2016	van Rijn, PCJ & Wäckers, FL	Journal of Applied Ecology	165	[36]
Benefits and Risks of Intercropping for Crop Resilience and Pest Management	2022	Huss, CP et al.	Journal of Economic Entomology	169	[37]
Perennial, species-rich wildflower strips enhance pest control and crop yield	2016	Tschumi, M et al.	Agriculture Ecosystems & Environment	162	[38]

**Table 3:** The most productive and most influential authors in the field of biological control with arthropod natural enemies.

**Rank**	**Author**	**Articles**	**Average citation per article**
1	Desneux, Nicolas	102	39.67
2	Duan, Jian J	46	13.15
3	Fathipour, Yaghoub	44	11.61
4	Urbaneja, Alberto	43	34.86
5	Biondi, Antonio	41	63.10
6	Lu, Yanhui	37	16.16
7	Wyckhuys, Kris	37	28.51
8	Daane, Kent M	31	29.74
9	Zang, Lian-Sheng	31	32.58
10	Kenis, Marc	30	66.77
Highly productive authors			
**Rank**	**Author**	**Citations (excluding self-citations)**	**Average citation per article**
1	Desneux, Nicolas	4641	44.63
2	Biondi, Antonio	2870	66.74
3	Tscharntke, Teja	2378	139.88
4	Rusch, Adrien	2287	91.48
5	Entling, Martin	2149	82.65
6	Landis, Douglas A.	2067	147.64
7	Kenis, Marc	2040	61.82
8	Martin, Emily A. P.	2000	153.85
8	Bommarco, Riccardo	1783	137.15
10	Urbaneja, Alberto	1722	40.05
Highly influential authors			

## Data Availability

The data that support the findings of this study are available from the corresponding author upon reasonable request.

## Supplementary Materials

The following supporting information can be downloaded at: https://www.mdpi.com/article/10.3390/insects17060609/s1, Table S1. Highly productive journals with 10 publications in the study domain of arthropod natural enemies in biological control. Table S2. Organizations excelling in the number of papers focusing on biological control with arthropod natural enemies. Table S3. Top 10 countries leading in published publications on biological control with arthropod natural enemies. Table S4. Publication output normalized by population and GDP (PPP) for the top 10 most productive countries. Table S5. The most supporting funding agencies in research on biological control with arthropod natural enemies. Table S6. Funding intensity normalized by GDP (PPP) for major funding countries/agencies (based on number of funded articles from Table S3). Table S7. Average publication year for the 30 most frequent keywords in the field of arthropod natural enemies in biological control (2016–2025). Table S8. References used in analysis. Table S9. Cluster validation by silhouette analysis: assessing cohesion and separation of co-citation networks in the field of arthropod natural enemies in biological control (2016–2025). Figure S1. The PRISMA (Preferred Reporting Items for Systematic Reviews and Meta-Analysis) flowchart illustrating the inclusion and exclusion criteria with corresponding literature search results (in parentheses). Figure S2. Author collaboration co-occurrence network in the field of arthropod natural enemies in biological control (2016–2025). Figure S3. Country collaboration co-occurrence map in the field of arthropod natural enemies in biological control (2016–2025). Figure S4. Keyword co-occurrence network and overlay visualization in the field of arthropod natural enemies in biological control (2016–2025). Figure S5. Top 25 keywords with the strongest citation bursts in the field of arthropod natural enemies in biological control (2016–2025). Figure S6. Co-citation clustering map of cited references in the field of arthropod natural enemies in biological control (2016–2025).
