# Supplementary material for: Arthropod Natural Enemies in Biological Control: A Systematic Bibliometric Analysis 2016–2025
Source: Insects. 2026 Jun 9;17(6):609. doi: 10.3390/insects17060609 (PMC13300250; doi:10.3390/insects17060609)
Supplement: Supplementary file 1 [file insects-17-00609-s001.zip › insects-4313629-supplementary.pdf]

Supplementary Materials

Supplementary Tables

TABLE S1 Highly productive journals with 10 publications in the study domain of arthropod natural enemies in biological control.

Journal	Number of articles	Citations to articles	Publisher	Impact factor (2024)	CiteScore
Biological Control	455	6731	Elsevier	3.4	7.9
Insects	430	430	MDPI	2.9	5.6
BioControl	208	208	Springer Nature	2.7	5.0
Journal of Economic Entomology	196	196	Oxford University Press	2.4	4.2
Pest Management Science	195	195	Wiley	3.8	7.8
Journal of Pest Science	186	186	Springer Nature	4.1	10.3
Agriculture Ecosystems& Environment	159	159	Elsevier	6.4	12.5
Biocontrol Science and Technology	140	140	Taylor & Francis Ltd	1.2	3.0
Environmental Entomology	138	138	Oxford University Press	1.5	3.6
Crop Protection	106	106	Elsevier	2.5	5.40

TABLE S2 Organizations excelling in the number of papers focusing on biological control with arthropod natural enemies.

Organizations	Country	Articles	% of total articles	citations
United States Department of Agriculture (USDA)	United States	537	8.12	11958
INRAE	France	363	5.49	12664
Centre National de la Recherche Scientifique (CNRS)	France	243	3.67	9420
University of California System	United States	238	3.60	7443
Chinese Academy of Agricultural Sciences (CAAS)	Peoples R China	218	3.30	6517
State University System of Florida	United States	198	2.99	2703
Empresa Brasileira de Pesquisa Agropecuaria (EMBRAPA)	Brazil	177	2.68	2318
University of Florida	United States	173	2.61	2703
Universite Cote d'Azur	France	162	2.45	7335
Universidade Federal de Vicosa	Brazil	152	2.41	3813

TABLE S3 Top 10 countries leading in published publications on biological control with arthropod natural enemies.

Rank	Country	No. of articles	% of total articles	Total citations	Total link Strength	Average publication year
1	USA	1604	24.24	16746	1129272	2020.45
2	Peoples R China	1116	16.87	10961	772977	2021.35
3	Brazil	770	11.64	7406	539020	2020.52
4	France	540	8.16	11476	748112	2020.42
5	Spain	427	6.45	6400	438377	2020.78
6	Italy	395	5.97	9027	476879	2020.92
7	Australia	328	4.96	5385	384708	2020.72
8	England	296	4.47	6601	391374	2020.66
9	Germany	290	4.38	6365	426652	2020.42
10	Canada	278	4.20	4878	300049	2020.65

TABLE S4. Publication output normalized by population and GDP (PPP) for the top 10 most productive countries.

Country	Total publications	Population (millions, 2024)	Publications per million	GDP PPP (billion USD, 2024)	Publications per billion USD GDP
United States	1,604	341.1[1]	4.70	29,298[2]	0.055
China	1,116	1,408.3[3]	0.79	38,209[2]	0.029
Brazil	770	212.6[4]	3.62	4,736[5]	0.163
France	540	68.6[6]	7.87	4,286[7]	0.126
Spain	427	48.6[8]	8.79	2,836[9]	0.151
Italy	395	58.9[10]	6.71	3,605[11]	0.110
Australia	328	27.4[12]	11.97	1,961[13]	0.167
England	296	57.1[14]	5.18	4,137[15]	0.072
Germany	290	83.6[16]	3.47	6,143[17]	0.047
Canada	278	41.3[18]	6.73	2,667[19]	0.104

Note: [1] United States population: U.S. Census Bureau, 2024 estimate (341.1 million as of July 1, 2024).

[2] United States and China GDP PPP: Worldometer / World Bank data (2024).

[3] China population: National Bureau of Statistics of China, 1,408.28 million (2024).

[4] Brazil population: IBGE (Brazilian Institute of Geography and Statistics), July 1, 2024 estimate (212.6 million).

[5] Brazil GDP PPP: CEIC Data / IMF estimate for 2024.

[6] France population: INSEE, as of January 1, 2025 (68.6 million; representing 2024 annual estimate).

[7] France GDP PPP: CEIC Data / IMF estimate for 2024.

[8] Spain population: Spanish National Statistics Institute (INE), 2024 estimate (48.6 million).

[9] Spain GDP PPP: CEIC Data / IMF estimate for 2024.

[10] Italy population: ISTAT, 2024 estimate (58.9 million).

[11] Italy GDP PPP: CEIC Data / IMF estimate for 2024.

[12] Australia population: Australian Bureau of Statistics (ABS), December 31, 2024 (27.4 million).

[13] Australia GDP PPP: CEIC Data / IMF estimate for 2024.

[14] England population: Office for National Statistics (ONS), mid-2024 estimate (57.1 million).

[15] United Kingdom GDP PPP (England included within UK): IMF estimate for 2024.

[16] Germany population: Destatis (Federal Statistical Office), December 31, 2024 (83.6 million).

[17] Germany GDP PPP: CEIC Data / IMF estimate for 2024.

[18] Canada population: Statistics Canada, July 1, 2024 (41.3 million).

[19] Canada GDP PPP: CEIC Data / IMF estimate for 2024. GDP PPP values are expressed in current international dollars (constant 2024 USD). Publications per million and per billion USD are calculated using the total publication counts from Table S3. Switzerland, despite high per-capita output, does not appear in the top 10 absolute publication ranking and is therefore not included in this table. For a complete list of normalized indicators including Switzerland, please refer to the full dataset available from the corresponding author.

TABLE S5 The most supporting funding agencies in research on biological control with arthropod natural enemies.

Funding Agency	Number of articles	% of total articles
National Natural Science Foundation of China (NSFC)	479	7.24
Conselho Nacional De Desenvolvimento Cientifico E Tecnologico (CNPQ)	390	5.89
Coordenacao De Aperfeicoamento De Pessoal De Nivel Superior (CAPES)	350	5.29
United States Department of Agriculture (USDA)	339	5.12
European Union (EU)	273	4.13
Spanish Government	191	2.89
National Key Research Development Program of China	121	1.83
National Science Foundation (NSF)	118	1.78
Fundacao De Amparo A Pesquisa Do Estado De Minas Gerais Fapemig	109	1.65
UK Research Innovation (UKRI)	107	1.64

TABLE S6. Funding intensity normalized by GDP (PPP) for major funding countries/agencies (based on number of funded articles from Table S3).

Funding agency	Country	Number of funded articles	GDP PPP (trillion USD, 2024)	Articles per trillion USD GDP
National Natural Science Foundation of China (NSFC)	China	479	38.15	12.6
Conselho Nacional De Desenvolvimento Cientifico E Tecnologico (CNPQ)	Brazil	390	4.74	82.3
Coordenacao De Aperfeicoamento De Pessoal De Nivel Superior (CAPES)	Brazil	350	4.74	73.8
United States Department of Agriculture (USDA)	USA	339	29.30	11.6
European Union (EU)	EU	273	28.04	9.7
Spanish Government	Spain	191	2.68	71.3
National Key Research Development Program of China	China	121	38.15	3.2
National Science Foundation (NSF)	USA	118	29.30	4.0
Fundacao De Amparo A Pesquisa Do Estado De Minas Gerais (Fapemig)	Brazil	109	4.74	23.0
UK Research Innovation (UKRI)	United Kingdom	107	4.28	25.0

*Approximate GDP PPP for the European Union as a whole (Eurostat/World Bank). Note: For agencies that fund research across multiple countries, GDP is approximated by the home country or regional aggregate. These values are intended as indicative comparisons of research intensity.

TABLE S7. Average publication year for the 30 most frequent keywords in the field of arthropod natural enemies in biological control (2016-2025).

Keyword	Occurrences	Average publication year
biological control	2,845	2018.2
natural enemy	1,876	2018.4
integrated pest management	1,234	2018.9
biodiversity	987	2019.1
conservation biological control	654	2020.3
Tuta absoluta	432	2020.7
Halyomorpha halys	389	2021.1
climate change	276	2021.8

TABLE S8**.** References used in analysis.

cluster	label	url
1	woodcock (2016)	https://doi.org/10.1016/j.agee.2016.06.023
1	vidal (2018)	https://doi.org/10.1111/ele.12874
1	van rijn (2016)	https://doi.org/10.1111/1365-2664.12605
1	tschumi (2016b)	https://doi.org/10.1111/1365-2664.12653
1	tschumi (2016a)	https://doi.org/10.1016/j.agee.2016.01.001
1	tscharntke (2016)	https://doi.org/10.1016/j.biocon.2016.10.001
1	tena (2016)	https://doi.org/10.1016/j.cois.2016.02.008
1	tamburini (2016)	https://doi.org/10.1111/1365-2664.12544
1	snyder (2019)	https://doi.org/10.1016/j.biocontrol.2019.04.017
1	rusch (2016)	https://doi.org/10.1016/j.agee.2016.01.039
1	redlich (2018)	https://doi.org/10.1111/1365-2664.13126
1	noriega (2018)	https://doi.org/10.1016/j.baae.2017.09.006
1	michalko (2019)	https://doi.org/10.1111/geb.12927
1	mccravy (2018)	https://doi.org/10.3390/insects9040170
1	martin (2019)	https://doi.org/10.1111/ele.13265
1	martin (2016)	https://doi.org/10.1890/15-0856
1	karp (2018)	https://doi.org/10.1073/pnas.1800042115
1	jeanneret (2021)	https://doi.org/10.1007/s10980-021-01248-0
1	huss (2022)	https://doi.org/10.1093/jee/toac045
1	holland (2016)	https://doi.org/10.1002/ps.4318
1	hatt (2018)	https://doi.org/10.1016/j.scitotenv.2017.11.296
1	han (2022)	https://doi.org/10.1146/annurev-ento-060121-060505
1	haan (2020)	https://doi.org/10.1016/j.tree.2019.10.003
1	gurr (2017)	https://doi.org/10.1146/annurev-ento-031616-035050
1	greenop (2018)	https://doi.org/10.1002/ecy.2378
1	gontijo (2019)	https://doi.org/10.1016/j.biocontrol.2018.10.014
1	ganser (2019)	https://doi.org/10.1016/j.agee.2019.02.010
1	fahrig (2017)	https://doi.org/10.1146/annurev-ecolsys-110316-022612
1	dunn (2020)	https://doi.org/10.1002/ps.5807
1	dassou (2016)	https://doi.org/10.1002/ece3.1917
1	dainese (2017b)	https://doi.org/10.1038/s41598-017-08316-z
1	dainese (2017a)	https://doi.org/10.1111/1365-2664.12747
1	campbell (2017)	https://doi.org/10.3390/insects8030101
1	calvo-agudo (2019)	https://doi.org/10.1073/pnas.1904298116
1	boesing (2017)	https://doi.org/10.1007/s10980-017-0503-1
1	benelli (2017)	https://doi.org/10.1007/s10340-017-0835-2
1	begg (2017)	https://doi.org/10.1016/j.cropro.2016.11.008
1	bartual (2019)	https://doi.org/10.1016/j.agee.2019.04.009
1	baillod (2017)	https://doi.org/10.1111/1365-2664.12910
1	aguilera (2020)	https://doi.org/10.1111/1365-2664.13712
2	stacconi (2019)	https://doi.org/10.1007/s10526-018-09914-0

2	soares (2019)	https://doi.org/10.1007/s10340-018-01074-5
2	rabiey (2019)	https://doi.org/10.1007/s10658-019-01814-y
2	quesada-moraga (2024)	https://doi.org/10.1007/s10340-023-01622-8
2	mouden (2017)	https://doi.org/10.1002/ps.4531
2	mills (2016)	https://doi.org/10.1016/j.biocontrol.2015.05.006
2	mazzetto (2016)	https://doi.org/10.1007/s10340-016-0746-7
2	mansour (2018)	https://doi.org/10.1127/entomologia/2018/0749
2	macias-rodriguez (2020)	https://doi.org/10.1016/j.micres.2020.126552
2	lovett (2018)	https://doi.org/10.1002/ps.4734
2	lee (2019)	https://doi.org/10.1093/jipm/pmz012
2	klieber (2016)	https://doi.org/10.1111/jen.12287
2	horowitz (2020)	https://doi.org/10.1007/s10340-020-01210-0
2	hill (2017b)	https://doi.org/10.7717/peerj.4179
2	haye (2016)	https://doi.org/10.1007/s10340-016-0737-8
2	han (2019)	https://doi.org/10.1007/s10340-018-1062-1
2	giunti (2022)	https://doi.org/10.1016/j.biocontrol.2022.105071
2	giorgini (2019)	https://doi.org/10.1007/s10340-018-01068-3
2	ferreira (2021)	https://doi.org/10.1007/s11274-021-03058-7
2	fernandes (2016)	https://doi.org/10.1016/j.chemosphere.2016.04.115
2	douglas (2016)	https://doi.org/10.7717/peerj.2776
2	desneux (2022)	https://doi.org/10.1007/s10340-021-01442-8
2	campos (2017)	https://doi.org/10.1007/s10340-017-0867-7
2	bueno (2017)	https://doi.org/10.1590/0103-8478cr20160829
2	brugger (2019)	https://doi.org/10.1038/s41598-019-44709-y
2	biondi (2018)	https://doi.org/10.1146/annurev-ento-031616-034933
2	biondi (2016)	https://doi.org/10.1007/s10526-015-9700-5
2	bamisile (2021)	https://doi.org/10.3389/fpls.2021.741804
2	aristizabal (2016)	https://doi.org/10.3390/insects7010006
2	ahmad (2021)	https://doi.org/10.1007/s10482-021-01577-9
3	zhang (2017)	https://doi.org/10.1007/s10340-017-0863-y
3	stouthamer (2017)	https://doi.org/10.1111/afe.12215
3	stahl (2019)	https://doi.org/10.1007/s10340-018-1061-2
3	roy (2016)	https://doi.org/10.1007/s10530-016-1077-6
3	pearson (2016)	https://doi.org/10.1016/j.biocon.2016.02.029
3	leskey (2018)	https://doi.org/10.1146/annurev-ento-020117-043226
3	kenis (2017)	https://doi.org/10.1007/s10530-017-1414-4
3	hui (2016)	https://doi.org/10.1007/s10530-016-1076-7
3	hill (2017a)	https://doi.org/10.4102/abc.v47i2.2152
3	heimpel (2018)	https://doi.org/10.1007/s10526-017-9841-9
3	hedstrom (2017)	https://doi.org/10.1007/s10340-017-0892-6
3	hajek (2016)	https://doi.org/10.1007/s10530-016-1075-8
3	garcia (2020)	https://doi.org/10.3390/insects11100662
3	duan (2018)	https://doi.org/10.3390/f9030142
3	dieckhoff (2017)	https://doi.org/10.1007/s10340-017-0868-6

3	conti (2021)	https://doi.org/10.1111/eea.12967
3	brockerhoff (2017)	https://doi.org/10.1007/s10530-017-1514-1
3	brewer (2017)	https://doi.org/10.1093/jee/tox204
3	bowling (2016)	https://doi.org/10.1093/jipm/pmw011
3	barratt (2018)	https://doi.org/10.1007/s10526-017-9831-y
3	bajwa (2016)	https://doi.org/10.1007/s00425-016-2510-x
3	abram (2017)	https://doi.org/10.1007/s10340-017-0891-7
3	abram (2016)	https://doi.org/10.1016/j.biocontrol.2016.04.002
4	zhou (2024)	https://doi.org/10.1021/acsomega.4c06628
4	turlings (2018)	https://doi.org/10.1146/annurev-ento-020117-043507
4	sisay (2019)	https://doi.org/10.3390/insects10070195
4	sisay (2018)	https://doi.org/10.1111/jen.12534
4	romeis (2019)	https://doi.org/10.1016/j.biocontrol.2018.10.001
4	reynolds (2016)	https://doi.org/10.3389/fpls.2016.00744
4	peterson (2016)	https://doi.org/10.3389/fpls.2016.01794
4	paredes-sanchez (2021)	https://doi.org/10.3390/molecules26185587
4	mitchell (2016)	https://doi.org/10.3389/fpls.2016.01132
4	kenis (2023)	https://doi.org/10.1127/entomologia/2022/1659
4	kenis (2019)	https://doi.org/10.3390/insects10040092
4	dai (2019)	https://doi.org/10.1111/pbi.13091
4	bakhat (2018)	https://doi.org/10.1016/j.cropro.2017.10.008
4	assefa (2019)	https://doi.org/10.1080/23311932.2019.1641902
4	agboyi (2020)	https://doi.org/10.3390/insects11020068
4	aartsma (2017)	https://doi.org/10.1111/nph.14475
5	zang (2021)	https://doi.org/10.1146/annurev-ento-060120-091620
5	wang zhi-zhi (2019)	https://doi.org/10.1016/s2095-3119(18)62078-7
5	van lenteren (2018)	https://doi.org/10.1007/s10526-017-9801-4
5	tougeron (2020)	https://doi.org/10.1111/een.12792
5	reineke (2016)	https://doi.org/10.1007/s10340-016-0761-8
5	li (2016)	https://doi.org/10.1146/annurev-ento-010715-023622
5	iost filho (2020)	https://doi.org/10.1093/jee/toz268
5	furlong (2017)	https://doi.org/10.1016/j.cois.2017.03.006
5	damien (2019)	https://doi.org/10.1016/j.cois.2019.07.002
5	castex (2018)	https://doi.org/10.1016/j.scitotenv.2017.11.027

TABLE S9. Cluster validation by silhouette analysis: assessing cohesion and separation of co-citation networks in the field of arthropod natural enemies in biological control (2016-2025).

Cluster	Publications	Average publication year	Silhouette	Average publication year
I	43	2017.86	0.977	pest regulation; agroforestry; biodiversity; ecosystem services; landscape ecology
II	38	2018.39	0.899	integrated pest management; indirect defense; plant volatiles; biological control
III	18	2017.92	0.721	biocontrol; chemical ecology; Hemiptera; invasive species; landscape management; pre-emptive; classical biological control
IV	12	2018.72	0.869	invasiveness; biotic; resistance; establishment; risk assessment
V	10	2019.72	0.891	biological control; egg parasitism; fall armyworm; invasive species; maize; Spodoptera frugiperda; Telenomus remus

Supplementary Figure Legends

FIGURE S1

The PRISMA (Preferred Reporting Items for Systematic Reviews and Meta-Analysis) flowchart illustrating the inclusion and exclusion criteria with corresponding literature search results (in parentheses).

FIGURE S2

Author collaboration co-occurrence network in the field of arthropod natural enemies in biological control (2016–2025).

FIGURE S3

Country collaboration co-occurrence map in the field of arthropod natural enemies in biological control (2016–2025).

FIGURE S4

Keyword co-occurrence network and overlay visualization in the field of arthropod natural enemies in biological control (2016–2025).

FIGURE S5

Top 25 keywords with the strongest citation bursts in the field of arthropod natural enemies in biological control (2016–2025).

FIGURE S6

Co-citation clustering map of cited references in the field of arthropod natural enemies in biological control (2016–2025).

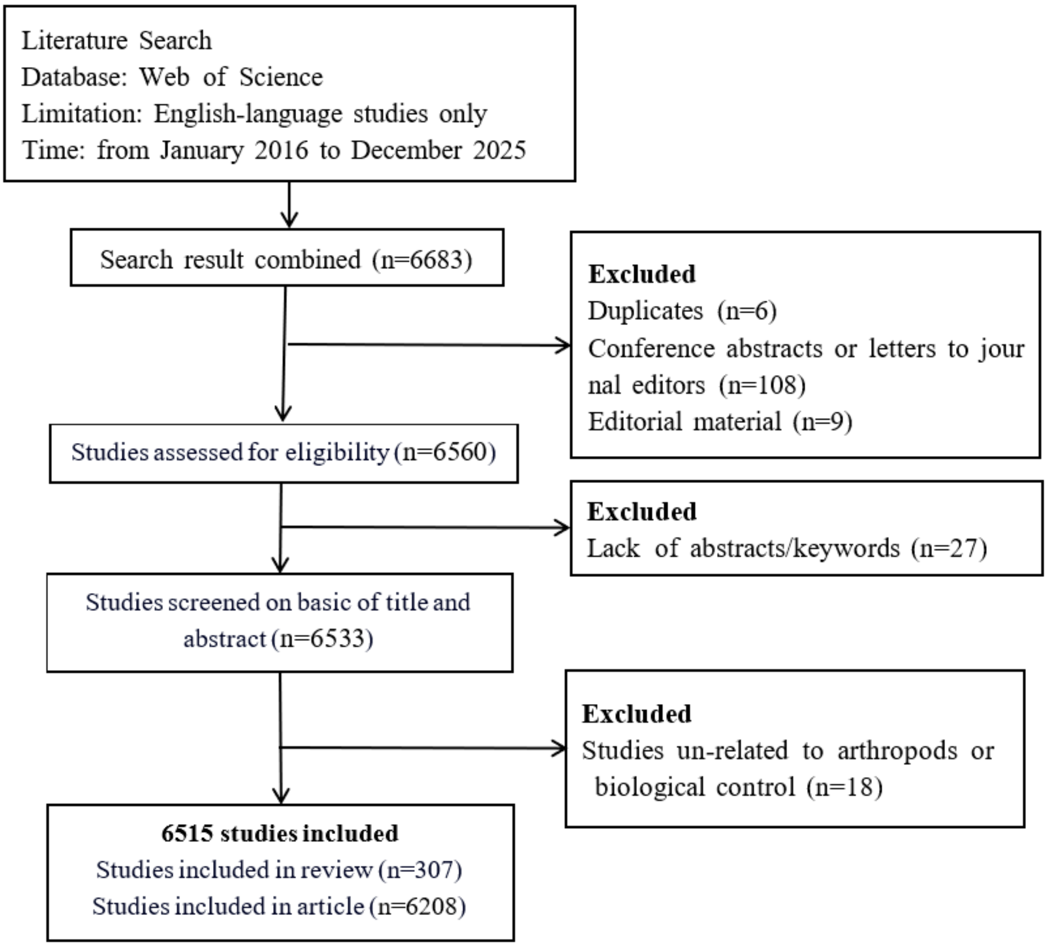

FIGURE S1

The PRISMA (Preferred Reporting Items for Systematic Reviews and Meta-Analysis) flowchart illustrating the inclusion and exclusion criteria with corresponding literature search results (in parentheses).

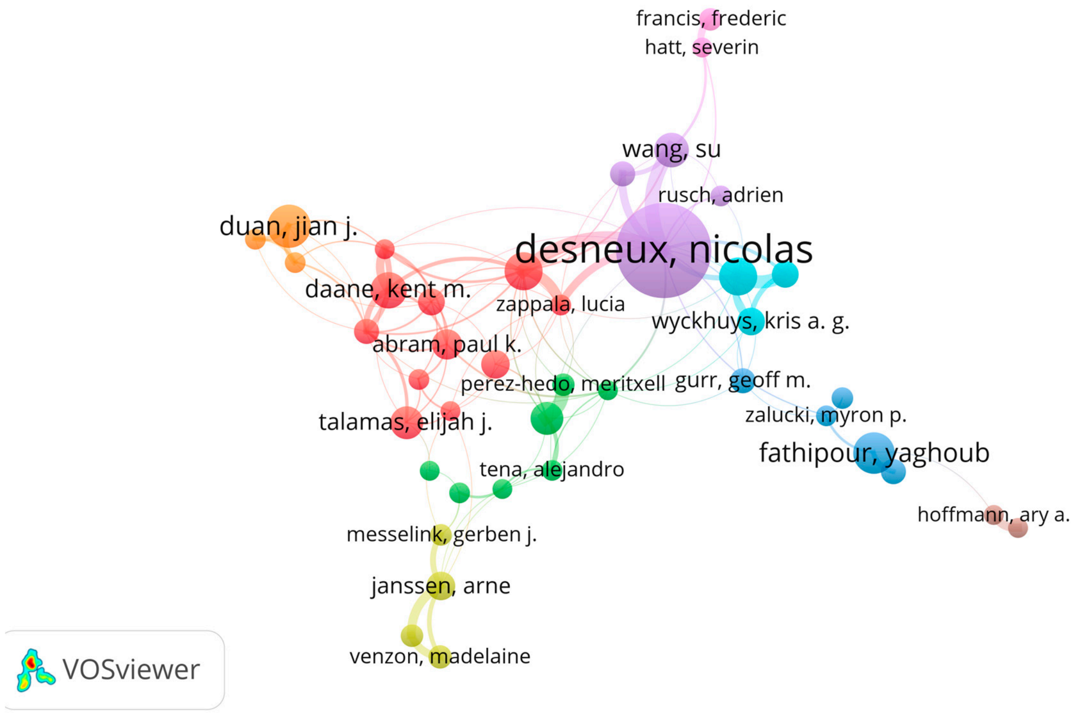

FIGURE S2

Author collaboration co-occurrence network in the field of arthropod natural enemies in biological control (2016–2025). This network was generated using VOSviewer v.1.6.19. The analysis was based on co-authorship relationships extracted from 6,515 publications retrieved from the Web of Science Core Collection. A minimum threshold of five publications per author was applied to ensure statistical reliability, and 331 authors met this criterion.

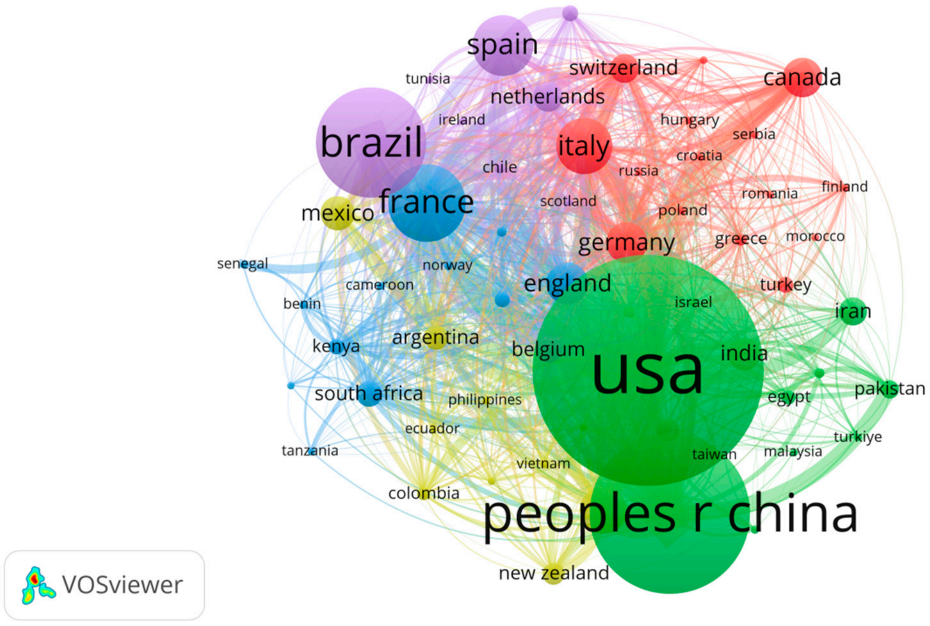

FIGURE S3

Country collaboration co-occurrence map in the field of arthropod natural enemies in biological control (2016–2025). This map was generated using VOSviewer v.1.6.19. Each node represents a country, and the node size is proportional to the number of publications from that country. The line (edge) between two countries indicates that they have co-authored at least one publication; thicker lines represent stronger collaboration (i.e., more co-authored papers). Node colors are determined by cluster assignment based on collaboration frequency: countries that co-author frequently are assigned to the same cluster and share the same color. Different colors distinguish different collaborative groups. The network layout is based on the VOS (Visualization of Similarities) algorithm, which positions countries with stronger collaboration relationships closer together.

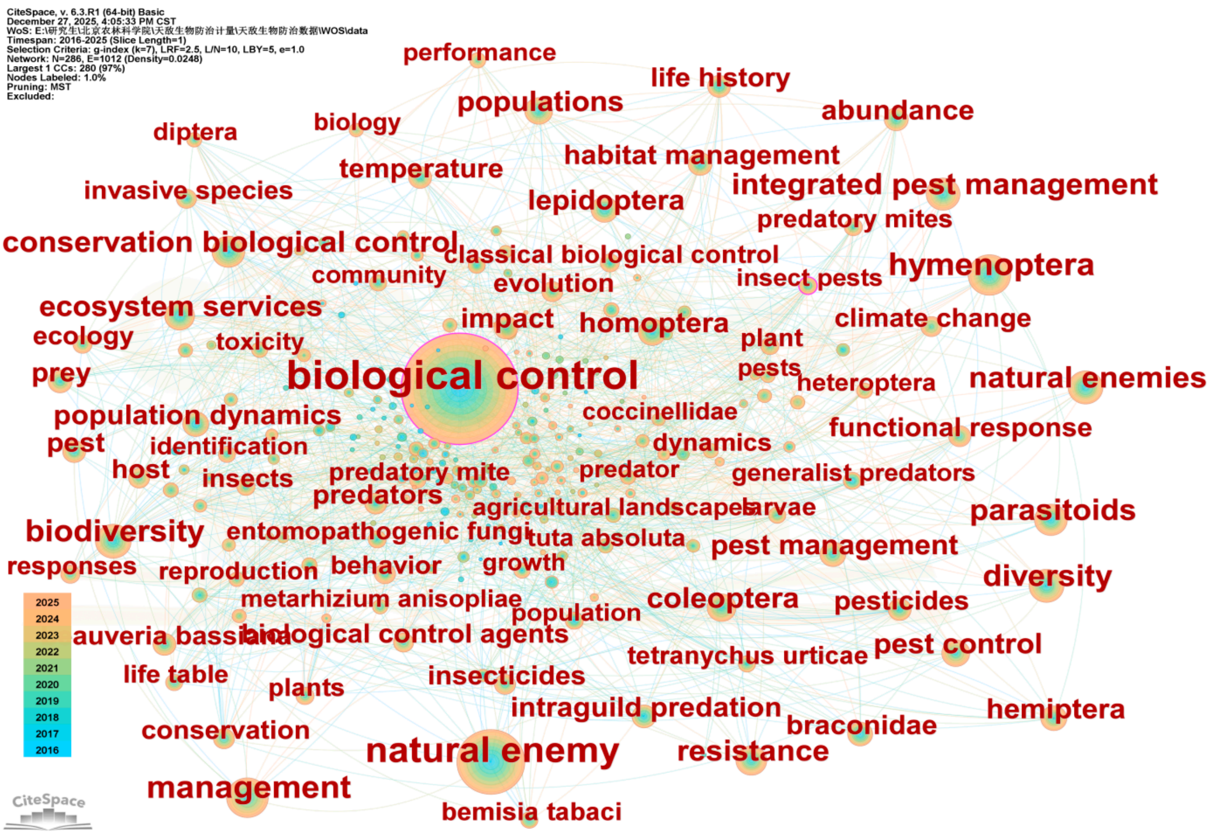

FIGURE S4

Keyword co-occurrence network and overlay visualization in the field of arthropod natural enemies in biological control (2016–2025). This network was generated using VOSviewer v.1.6.19. Each node (circle) represents a keyword. The node size is proportional to the frequency of that keyword in the literature. The line between two keywords represents a co-occurrence relationship, and the distance between nodes is inversely related to their co-occurrence frequency (i.e., keywords that appear together more often are positioned closer). In the overlay visualization mode, node colors represent the average publication year of documents containing that keyword, following a gradient: blue indicates earlier average publication year (ca. 2016 – 2018), green indicates intermediate years (ca. 2018 – 2021), and yellow/red indicates more recent average publication year (ca. 2022 – 2025). This color coding allows readers to identify “hot” (recent) vs. “cold” (older) research topics at a glance. The network is divided into clusters (groups of keywords with strong co-occurrence relationships), distinguished by different base colors in the network visualization mode. Only the top 200 most frequent keywords are shown for visual clarity.

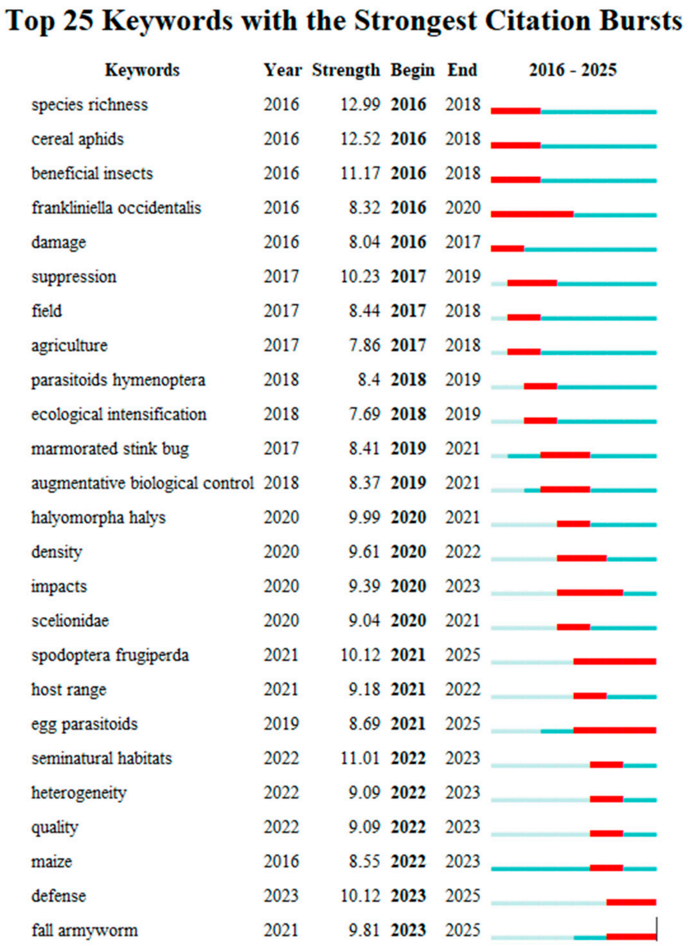

FIGURE S5

Top 25 keywords with the strongest citation bursts in the field of arthropod natural enemies in biological control (2016–2025). This burst was generated using CiteSpace 6.4.R1 with burst detection based on the Kleinberg algorithm (default parameters: γ = 0.8, burst duration range 2 – 5 years). For each keyword, the horizontal bar indicates its time interval within the study period. A blue bar represents the period during which the keyword appeared but had not yet experienced a burst. A red bar indicates the burst period- the specific time interval during which the keyword’ s frequency increased dramatically (statistically significant surge). The burst strength (a numerical value shown on the right of each keyword) measures the intensity of the burst; higher values indicate more dramatic increases in research attention. The timeline spans from 2016 to 2025. For example, “seminatural habitats” (burst strength 11.01) shows a red bar from 2022 to 2023, indicating that this keyword was a rapidly emerging research hotspot during that period.

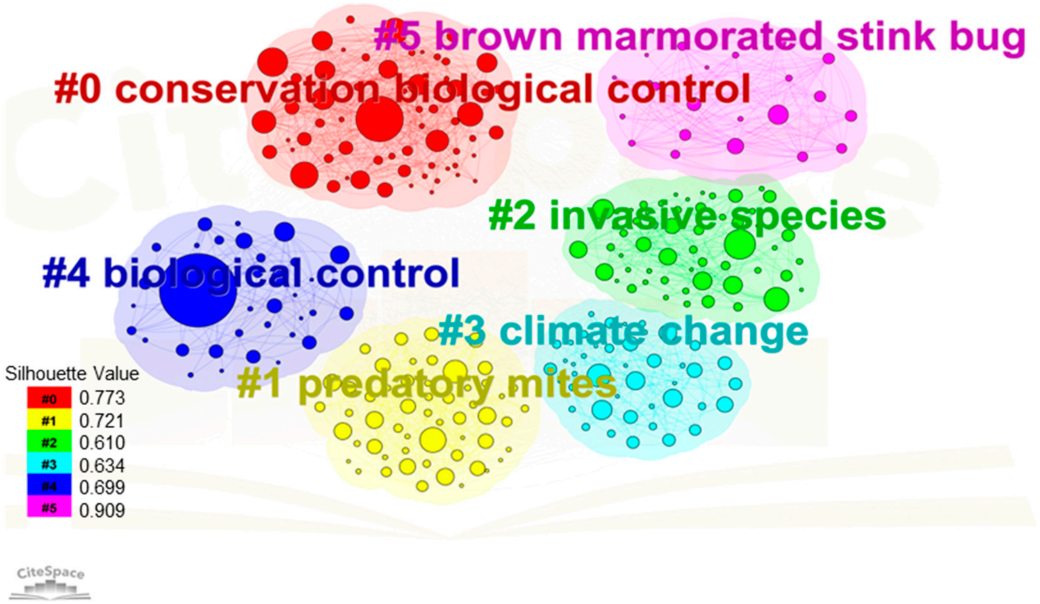

FIGURE S6

Co-citation clustering map of cited references in the field of arthropod natural enemies in biological control (2016–2025). This map was generated using CiteSpace 6.4.R1 (time span: 2016–2025, one-year time slice, g-index k = 7, no network pruning). Each numbered cluster (#0–#5) represents a group of cited references that are frequently co-cited together, indicating a shared research theme. The silhouette value (S value) for each cluster is shown in the inset table (e.g., #0: S = 0.773). The silhouette value measures the internal consistency or homogeneity of a cluster. It ranges from –1 to 1. A silhouette value > 0.7 indicates that the cluster is highly credible and well-defined, with strong similarity among its member references. Values between 0.5 and 0.7 are considered acceptable, while values below 0.5 suggest that the cluster may be poorly defined. All clusters in this map have silhouette values above 0.6, and most exceed 0.7, confirming that the clustering structure is statistically reliable. Modularity Q = 0.3875 (> 0.3) further indicates significant cluster structure. Node colors distinguish different clusters; the layout is based on the default CiteSpace algorithm, which positions highly co-cited references in close proximity.
